# Maternal exposure to Wenchuan earthquake and prolonged risk of offspring birth outcomes: a natural experiment study

**DOI:** 10.1186/s12884-020-03206-1

**Published:** 2020-09-22

**Authors:** Qiguo Lian, Jiaying Ni, Jun Zhang, Julian Little, Shan Luo, Lin Zhang

**Affiliations:** 1 NHC Key Lab. of Reproduction Regulation (Shanghai Institute of Planned Parenthood Research), Fudan University, Shanghai, 200237 China; 2 Obstetric and Gynecology Department, Xinhua Hospital, Shanghai Jiao Tong University School of Medicine, Shanghai, 200092 China; 3 MOE-Shanghai Key Lab of Children’s Environmental Health, Xinhua Hospital, Shanghai Jiao Tong University School of Medicine, Shanghai, 200092 China; 4 School of Epidemiology and Public Health, Faculty of Medicine, University of Ottawa, Ottawa, K1N 6N5 Canada; 5 West China Second University Hospital, Sichuan University, Chengdu, 610041 China; 6 Obstetrics Department, International peace maternity and child health hospital of China, Shanghai Jiao Tong University School of Medicine, 910 Hengshan Road, Shanghai, 200030 China

**Keywords:** Wenchuan earthquake, Adverse birth outcomes, Preterm birth

## Abstract

**Background:**

The prolonged effects of disasters on reproductive outcomes among the survivors are less studied, and the findings are inconsistent. We examined the associations of maternal exposure to the 2008 Wenchuan earthquake years before conception with adverse birth outcomes.

**Methods:**

We included 73,493 women who delivered in 96 hospitals in 24 provinces and autonomous regions from the 2015/16 China Labor and Delivery Survey. We weighted the multivariable logistic models based on the combination of coarsened exact matching (CEM) weight and survey weight, and performed sex-stratified analysis to test whether associations of maternal earthquake exposure with adverse birth outcomes (Stillbirth, preterm birth [PTB], low birthweight [LBW], and small for gestational age [SGA]) varied by sex.

**Results:**

The bivariate models showed that the weighted incidence of each adverse birth outcome was higher in exposed group than unexposed group: stillbirth (2.00% vs. 1.33%), PTB (14.14% vs. 7.32%), LBW (10.82% vs. 5.76%), and SGA (11.32% vs. 9.52%). The multivariable models showed maternal earthquake exposure was only associated significantly with a higher risk of PTB in offspring among all births (adjusted risk ratio [aRR](95%CI):1.25(1.06–1.48), *P* = 0.010). The sex-stratified analysis showed the association was significant among male births (aRR (95%CI): 1.40(1.12–1.75),*P* = 0.002),but unsignificant among female births. The sensitivity analysis reported similar findings.

**Conclusions:**

The 2008 Wenchuan earthquake exposure has a long-term effect on PTB. Maternal acute exposure to disasters could be a major monitor for long-term reproductive outcomes. More attention should be paid to the underlining reasons for disaster-related adverse birth outcomes.

## Background

Adverse birth outcomes, such as low birthweight (LBW), preterm birth (PTB) and small for gestational age (SGA), are associated with numerous risk factors, including not only maternal characteristics [1, 2] and environmental contaminants [3], but also external stressors [4]. There is growing evidence that maternal exposure to natural or manmade disasters, including major earthquakes, ice storms and terrorist attacks during pregnancy could impact the birth outcomes intensively [5–9]. However, most of the existing studies to date have focused on the women who were pregnant during or immediately after a disaster and leaving behind the exposed women who get pregnant later [10]. Hence, the long-term negative effects of maternal acute exposure to disasters on reproductive outcomes are less studied.

Moreover, the few existing studies of prolonged effects of disasters on reproductive outcomes among the survivors reported mixed results, with one showing negative effects among women exposed to the 2001 World Trade Center disaster in Unites States [10] and another showing no effects among the women experienced the 2011 Fukushima Daiichi nuclear disaster in Japan [11]. Thus, the long-term effects of disasters remain unclear and are worthy of further investigation.

The 8.0-magnitude Wenchuan earthquake hit Wenchuan, Sichuan province on May 12, 2008, leading to 69,197 deaths, 374,176 injuries and 18,222 missing persons [12]. The disaster was one of the biggest and deadliest earthquakes in Chinese history, it affected more than 15 million people and left 4.8 million people homeless [12]. Existing studies assessed the long-term effects of the earthquake on mental health [12] and puberty timing [13] among the adolescent survivors. No studies to date, however, have evaluated the long-term effects of 2008 Wenchuan earthquake on birth outcomes, although a hospital-based study showed increased ratios of LBW and PTB within the first 12 months post-disaster in 2 counties affected by Wenchuan earthquake [8].

To bridge the gap, we evaluated the data from a national hospital-based cross-sectional study to investigate the long-term effects of the Wenchuan earthquake on birth outcomes. We hypothesized that the women exposed to the devastating earthquake and became pregnant years later would be associated with elevated risks of adverse birth outcomes in offspring. Given that male births are more vulnerable to maternal stress [14], we, therefore, hypothesized that there are sex differences in these associations.

## Methods

### Study design and participants

Data in the present study were from the China Labor and Delivery Survey (CLDS), a multi-center, hospital-based cross-sectional survey throughout China to estimate the national labour and delivery information of new births between March 1, 2015, and December 31, 2016. Using a stratified multistage cluster sampling design, the CLDS selected 112 hospitals with at least 1000 childbirths per year from 25 provinces and autonomous regions across China mainland. Details on the sampling strategy of CLDS have been described elsewhere [15]. The CLDS established a data coordination center (DCC) to retrieve, review and extract the data of new births and their mothers from hospital records in the selected hospitals. Within each calendar year, the DCC randomly selected 6 weeks for hospitals with at least 6000 childbirths per year, or 10 weeks for hospitals with less than 6000 childbirths per year for data collection. Within each selected week, all births born at 24 weeks gestation or more or weighing at least 500 g were eligible for inclusion.

Although the CLDS was a cross-sectional observational study, the Wenchuan earthquake generated a natural experimental setting to investigate the long-term effects of the earthquake for birth outcomes among the female survivors who became pregnancy years following the devastating earthquake [13, 16]. In this natural experiment, the earthquake event that divided the participants into exposed and unexposed groups was unexpected and not under the control [17]. In the current analysis, 96 hospitals with 70% or higher response rates in 24 provinces and autonomous regions, including Sichuan province, were involved.

The Research Project Review Panel at the Department of Reproductive Health and Research of World Health Organization (WHO) reviewed and approved the protocol of the CLDS for technical content. The CLDS was approved by the WHO Research Ethics Review Committee and the ethics committees in all participating hospitals. Informed consent was not required, as the collected data from hospital records were anonymized and de-identified to preserve privacy for the participants.

### Exposure

In this natural experiment, individual-level maternal exposure to the 2008 Sichuan earthquake was unmeasured in the original design and is difficult to define, as noted in the previous review [18], indicator of the earthquake exposure was obtained from the location of the delivery hospitals. We labeled the participants who delivered in 5 hospitals of Sichuan province as a natural indicator of earthquake exposure, and their counterparts in 91 hospitals of other provinces as an unexposed group.

### Outcomes

The main outcomes of interest were the outcome of delivery, gestational age, birthweight, and birthweight for gestational age. The corresponding adverse birth outcomes included stillbirth, PTB, LBW, and SGA.

We defined stillbirth as the death of a baby before or during delivery after 23 weeks gestation or weighing at least 500 g at birth. We divided gestational age at birth as preterm (< 37 weeks), term (37–41 weeks) and post-term (≥42 weeks) birth. We classified birthweight into low (< 2500 g), normal (2500–3999 g) and high (≥4000 g) groups. We categorized sex-specific birth weight-for-gestational age as small (<10th percentile), appropriate (10th–90th percentile) and large (>90th percentile) categories.

### Covariates

We divided maternal age into young (< 20 years), reference (20–34 years) and advanced (≥35 years) class. We categorized maternal education attainment as low (junior school or below), middle (high school, technical school, or junior college), and high (bachelor degree or above) group. We labeled the women as residents if they held the hukou in the province where they delivered, or migrants if not. According to the WHO classification of body mass index (BMI, defined as weight in kilograms divided by the square of height in meters), we categorized maternal pre-pregnancy BMI as underweight (< 18.5 kg/m^2^), healthy weight (18.5–24.9 kg/m^2^), and overweight or obesity (≥25 kg/m^2^) [19].

Maternal medical conditions in the present study included maternal diseases (hypertension, gestational hypertension, diabetes, gestational diabetes, cardiac disease, renal disease, autoimmune disease, thyroid disease, gestational thyroid disease, gestational anemia, asthma and gestational asthma), history of PTB, method of conception, parity (primipara or multipara), multiple pregnancy or singleton pregnancy, mode of delivery and sex of newborns.

### Statistical analysis

Statistical analysis was performed from January 5, 2020, to March 12, 2020. Statistical analyses were performed using Stata/SE 15.1(StataCorp LLC, College Station, TX, United States). To obtain a national estimation of labour and delivery information, survey weight for each newborn included in CLDS was calculated using the delivery data from 2016 China Statistical Yearbook in the previous study [15]. The *svy* and *subpop* option were used to account for complex survey design.

We first examined distributions of the exposure indicator, outcome variables, and covariates, and investigated the associations of exposure indicator with outcome variables and covariates using χ^2^ tests, and found the distribution of a few covariates was not balanced between the two groups (Table 1). To account for pregnant women who delivery in earthquake-affected areas being inherently different from those who do not, we matched the data first and weighted the analyses based on coarsened exact matching, the global imbalance was measured by overall L1 statistic, ranging from 0 to 1, with larger value indicating larger imbalance between the two groups [20]. Candidate variables for the coarsened exact matching (CEM)–weighted model included hospital level and the covariates measured in the study (Table 1). We obtained new weight using multiplying the CEM weights by the survey weight, then declared the data using *svyset* command with the new weight. We reported CEM–weighted adjusted risk ratio (aRR) and 95% confidence interval (CI) using *adjrr* command [21] after weighted logistic regression.

**Table 1:** Descriptive Statistics of potential covariables among Wenchuan exposure and non-exposure group

	No. (Weighted %)				
Characteristic	Total	Exposure	Non-exposure	χ^2^	*P* value
Maternal age					
< 20 years	1093 (2.39)	93 (2.70)	1000 (2.38)	1.94	0.413
20–34 years	63,948 (86.41)	2933 (86.70)	61,015 (86.40)		
> 34 years	8364 (11.20)	386 (10.60)	7978 (11.22)		
Maternal education					
Low	16,763 (36.38)	911 (35.34)	15,852 (36.41)	9.83	< 0.001
Middle	29,302 (39.70)	1144 (44.18)	28,158 (39.56)		
High	19,846 (23.93)	529 (20.48)	19,317 (24.03)		
Hukou					
Local	62,833 (87.15)	3303 (96.73)	59,530 (86.81)	230.02	< 0.001
Migrant	10,150 (12.85)	101 (3.27)	10,049 (13.19)		
Pre-pregnant BMI					
Underweight	6769 (12.95)	266 (15.18)	6503 (12.90)	6.89	0.001
Healthy weight	39,463 (72.49)	1208 (73.37)	38,255 (72.47)		
Overweight/Obese	8105 (14.56)	184 (11.45)	7921 (14.64)		
History of preterm					
Yes	899 (1.47)	34 (1.04)	865 (1.48)	3.01	0.083
No	72,388 (98.53)	3370 (98.96)	69,018 (98.52)		
Artificial Conception					
Yes	4690 (6.47)	128 (3.33)	4562 (6.58)	50.10	< 0.001
No	68,491 (93.53)	3287 (96.67)	65,204 (93.42)		
Parity					
Primiparous	41,207 (47.37)	1790 (52.69)	39,417 (47.18)	28.09	< 0.001
Multiparous	32,062 (52.63)	1613 (47.31)	30,449 (52.82)		
Diabetes					
Yes	770 (0.90)	38 (0.98)	732 (0.90)	0.19	0.664
No	72,276 (99.10)	3368 (99.02)	68,908 (99.10)		
Heart disease					
Yes	278 (0.32)	21 (0.53)	257 (0.32)	2.78	0.095
No	72,998 (99.68)	3393 (99.47)	69,605 (99.68)		
Renal disease					
Yes	121 (0.13)	4 (0.10)	117 (0.13)	0.14	0.708
No	73,211 (99.87)	3410 (99.90)	69,801 (99.87)		
Autoimmune disease					
Yes	206 (0.16)	23 (0.69)	183 (0.14)	52.33	< 0.001
No	72,764 (99.84)	3391 (99.31)	69,373 (99.86)		
Hyperthyroidism					
Yes	270 (0.39)	15 (0.44)	255 (0.38)	0.19	0.663
No	72,661 (99.61)	3396 (99.56)	69,265 (99.62)		
Hypothyroidism					
Yes	991 (0.88)	34 (1.13)	957 (0.87)	1.72	0.190
No	71,922 (99.12)	3377 (98.87)	68,545 (99.13)		
Asthma					
Yes	57 (0.05)	2 (0.06)	55 (0.05)	0.12	0.732
No	73,232 (99.95)	3411 (99.94)	69,821 (99.95)		
Gestational Diabetes					
Yes	7372 (11.43)	173 (5.99)	7199 (11.63)	64.48	< 0.001
No	65,017 (88.57)	3234 (94.01)	61,783 (88.37)		
Gestational hypertension					
Yes	3415 (4.16)	149 (4.06)	3266 (4.17)	0.06	0.802
No	69,004 (95.84)	3233 (95.94)	65,771 (95.83)		
Gestational anemia					
Yes	9293 (13.26)	147 (4.90)	9146 (13.55)	167.22	< 0.001
No	64,029 (86.74)	3268 (95.10)	60,761 (86.45)		
Gestational hyperthyroidism					
Yes	300 (0.32)	10 (0.27)	290 (0.32)	0.20	0.658
No	72,636 (99.68)	3405 (99.73)	69,231 (99.68)		
Gestational hypothyroidism					
Yes	2024 (1.90)	58 (2.16)	1966 (1.90)	0.73	0.394
No	70,910 (98.10)	3357 (97.84)	67,553 (98.10)		
Gestational asthma					
Yes	45 (0.05)	1 (0.02)	44 (0.05)	0.69	0.407
No	73,350 (99.95)	3414 (99.98)	69,936 (99.95)		
Multiple pregnancy					
Yes	1569 (1.78)	105 (2.72)	1464 (1.75)	10.76	0.001
No	71,921 (98.22)	3311 (97.28)	68,610 (98.25)		
Mode of delivery					
Cesarean	27,096 (36.77)	1838 (54.67)	25,258 (36.14)	332.93	< 0.001
Vaginal	46,073 (63.23)	1574 (45.33)	44,499 (63.86)		
Infant sex					
Female	34,170 (47.30)	1636 (47.97)	32,534 (47.27)	0.45	0.504
Male	39,323 (52.70)	1780 (52.03)	37,543 (52.73)		

In sensitive analyses, we restricted our analysis to singleton births, given that multiple pregnancies carry significantly higher risks of birth outcomes, including preterm birth [22] and stillbirth [23]. We also performed sex-stratified analysis as we hypothesized sex differences in stress responses [14]. To minimize the effect of migration on earthquake exposure, we further excluded the migrants and assessed the associations among the local pregnant women only.

## Results

We drew a total of 73,493 hospital records in our primary analysis, including 34,170 female births (47.30%), 1569 multiple births (1.78%) and 10,150 pregnant migrant women (12.85%), more details of the delivery information can be found in Table 1. After matching with CEM method, there are 37,059 matched cases (33,812 in unexposed group, 3247 in exposed group). The overall L1 statistic reduced from 0.55616578 before matching to 1.733e-14 after matching, suggesting that our matching significantly improved the imbalance in covariates between the two groups.

The weighted incidences of adverse birth outcomes were 1.35% for stillbirth, 7.55% for PTB, 5.93% for LBW, and 9.58% for SGA in our sample (Table 2). As showed in Table 2, the incidence of each adverse birth outcome was higher in exposed group: stillbirth (2.00% vs 1.33%, *P* = 0.018), PTB (14.14% vs 7.32%, *P* < 0.001), LBW (10.82% vs 5.76%, *P* < 0.001), and SGA (11.32% vs 9.52%, *P* = 0.008).

**Table 2:** The incidence of outcomes of interest among Wenchuan exposure and non-exposure group

	No. (Weighted %)				
Birth outcome	Total	Exposure	Non-exposure	χ^2^	*P* value
Outcome of delivery					
Stillbirth	873 (1.35)	75 (2.00)	798 (1.33)	5.63	0.018
Livebirth	72,580 (98.65)	3340 (98.00)	69,240 (98.67)		
Weeks of gestation					
Preterm	6290 (7.55)	503 (14.14)	5787 (7.32)	75.82	< 0.001
Term	65,337 (92.01)	2808 (85.46)	62,529 (92.24)		
Post-term	250 (0.44)	17 (0.41)	233 (0.44)		
Birth weight					
Low	4859 (5.93)	392 (10.82)	4467 (5.76)	47.91	< 0.001
Normal	63,658 (87.44)	2833 (83.77)	60,825 (87.57)		
High	4728 (6.63)	174 (5.42)	4554 (6.67)		
Birth weight for gestational age					
Small	6877 (9.58)	394 (11.32)	6483 (9.52)	4.83	0.008
Appropriate	59,415 (80.86)	2716 (79.93)	56,699 (80.89)		
Large	6977 (9.56)	292 (8.75)	6685 (9.59)		

Maternal exposure to Wenchuan Earthquake years before pregnancy was associated with significantly higher of risk of PTB in offspring (CEM–weighted aRR, 1.25; 95% CI, 1.06–1.48; *P* = 0.010) among all births, and similar results were found in other subpopulation, including singleton births, all livebirths, and singleton livebirths (Tables 3 and 4). However, the associations of earthquake exposure with stillbirth, LBW, and SGA were all non-significant in four samples (Table 3 and Table 4). Associations of maternal earthquake exposure with PTB varied by sex in all birth and singleton births, with significant associations observed in male births but not in female births (Table 3).

**Table 3:** Associations of maternal exposure to Wenchuan earthquake with adverse birth outcomes among all births

	Adjusted Risk Ratio^a^ (95% Confidence Interval, *P* value)	
Birth outcome	Model 1 (All births)	Model 2 (Singleton births)
Total		
Stillbirth	1.21 (0.71–2.04, 0.484)	1.29 (0.75–2.23, 0.359)
Preterm birth	1.25 (1.06–1.48, 0.010)	1.22 (1.02–1.47, 0.033)
Low birth weight	1.08 (0.89–1.31, 0.430)	1.03 (0.84–1.28, 0.755)
Small for gestational age	0.93 (0.71–1.22, 0.583)	0.91 (0.68–1.21, 0.524)
Girls		
Stillbirth	1.14 (0.58–2.26, 0.705)	1.28 (0.63–2.63, 0.494)
Preterm	1.09 (0.84–1.41, 0.535)	1.07 (0.81–1.42, 0.638)
Low birth weight	1.00 (0.76–1.32, 0.991)	0.98 (0.72–1.33, 0.897)
Small for gestational age	0.85 (0.55–1.33, 0.489)	0.84 (0.53–1.35, 0.474)
Boys		
Stillbirth	1.25 (0.57–2.75, 0.579)	1.28 (0.57–2.86, 0.547)
Preterm	1.40 (1.12–1.75, 0.002)	1.36 (1.07–1.73, 0.012)
Low birth weight	1.17 (0.89–1.53, 0.253)	1.09 (0.82–1.47, 0.547)
Small for gestational age	1.03 (0.84–1.26, 0.788)	1.01 (0.82–1.24, 0.943)

^a^Adjusted maternal diseases, history of PTB, method of conception, parity, multiple pregnancy or singleton pregnancy, mode of delivery and sex of newborns

**Table 4:** Associations of maternal exposure to Wenchuan earthquake with adverse birth outcomes among livebirths

	Adjusted Risk Ratio^a^ (95% Confidence Interval, *P* value)	
Birth outcome	Model 1 (All livebirths)	Model 2 (Singleton livebirths)
Total		
Preterm birth	1.27 (1.06–1.51, 0.009)	1.24 (1.02–1.50, 0.031)
Low birth weight	1.09 (0.89–1.33, 0.427)	1.02 (0.81–1.27, 0.857)
Small for gestational age	0.91 (0.69–1.20, 0.505)	0.89 (0.66–1.19, 0.417)
Girls		
Preterm	1.06 (0.81–1.40, 0.675)	1.04 (0.77–1.41, 0.798)
Low birth weight	0.98 (0.73–1.31, 0.871)	0.93 (0.67–1.29, 0.676)
Small for gestational age	0.85 (0.54–1.35, 0.499)	0.83 (0.52–1.34, 0.451)
Boys		
Preterm	1.47 (1.18–1.84, 0.001)	1.43 (1.12–1.82, 0.004)
Low birth weight	1.22 (0.93–1.61, 0.154)	1.13 (0.83–1.54, 0.420)
Small for gestational age	0.99 (0.80–1.22, 0.913)	0.96 (0.78–1.20, 0.738)

^a^Adjusted maternal diseases, history of PTB, method of conception, parity, multiple pregnancy or singleton pregnancy, mode of delivery and sex of newborns

The analyses that focused on the local residents yielded similar results. Although maternal earthquake exposure was not associated with most of the adverse birth outcomes, it related to increased risk of PTB, and the associations were also sex differential (eTable 1 in the supplement).

## Discussion

This, to our best knowledge, is the first study to investigate the long-effect of the Wenchuan earthquake on birth outcomes among the survivors that became pregnancy years later. The present study retrospectively compared the risks of 5 birth outcomes, including stillbirth, LBW, and SGA, from 2015 to 2016 at the national level between the Wenchuan earthquake-affected and unaffected areas. The results showed the incidence of 4 adverse birth outcomes were all higher in earthquake exposed group. After reducing the imbalance between the exposed and unexposed group based on CEM method, we observed the long-term effects of maternal earthquake exposure on PTB among male births only. The findings mentioned above still held within singleton births, all livebirth, and singleton livebirths, and remained unaffected by maternal migrant status.

Previous studies on disaster-related short-term adverse birth outcomes have produced mixed results between different earthquakes, with some studies, including one in Sichuan, China, showing increased risks of LBW, PTB, and SGA [7, 8, 24], while others studies suggesting nonsignificant changes in adverse birth outcomes [25, 26]. Furthermore, the findings on the same disasters, Hurricane Katrina for instance, have also been inconsistent [27, 28], indicating further research is likely needed to understand the effects on birth outcomes.

The few existing studies of natural and manmade disasters also turned up conflicting evidence of their long-term effects on adverse birth outcomes. One study on the 2001 World Trade Center disaster indicated that the effects of 11 September attacks on reproductive outcomes may last within the first 2 years, and reduce to zero in 3 years or later [10]. However, another study on the 2011 Fukushima Daiichi nuclear disaster showed no changes in birth outcomes within the 3 years following the Fukushima disaster [11]. Our findings suggested that Wenchuan earthquake increases the risks of PTB, but not all adverse birth outcomes, among the birth 7 years after the disaster, and female births seem to be immune to the earthquake exposure.

While it is difficult to compare the findings from different disasters directly, joint consideration of the commonalities may help to understand the mechanisms underlying disaster-related adverse birth outcomes [10]. Disaster-related adverse birth outcomes are associated with male vulnerability in utero perturbations,which could possiblely explained by genetic contributions [29]. The sex-specific X-linked genes expression in placenta may confer protection by fostering adaptability to survive [29, 30].

Disaster-related stress is another potential pathway from disaster occurrence to adverse birth outcomes [31]. Preterm birth, in evolutionary context, can be understood as a predictive adaptive response to the perceived deprived environment to increase the chances of survival and reproduction [32, 33]. Maternal psychosocial stress, including post-traumatic stress, could serve as an independent trigger for PTB [18, 29], given that a previous study of 1986 Chernobyl accident identified radiation-related anxiety as the prime culprit of PTB [34] and Wenchuan earthquake-related posttraumatic stress symptoms remained especially common among female survivors 5 years after the earthquake [35]. Disaster-related stress, in our opinion, could be main potential factor behind the long-term adverse effects of Wenchuan earthquake exposure on birth outcomes. The heightened maternal stress hormones, including glucocorticoids, may service an important biological mechanism behind the long-lasting earthquake-related adverse birth outcomes [36].

Our study has important implications for policy and clinical practice. Policy makers should acknowledge the long-lasting adverse effects of the earthquake on reproduction health in post-disaster recovery and invest long-term psychological support for the female survivors. Obstetricians also could reduce the risks of adverse birth outcomes by identifying the pregnant women experienced earthquake and providing targeted psychological interventions.

### Limitations

There are some important limitations should be noted. First, the exposure assessment was defined by the geographical location of the delivery hospitals, hence we restricted to the pregnant women with Sichuan hukou in stratified analysis to make sure exposure group actually experienced the disaster. Second, although we have adjusted for the confounders available in the dataset, the between-group comparability may still compromise the validity of our findings, given that some inherent non-comparability between groups was less likely to removed. Difference in difference analysis (before-after earthquake comparision with a parallel design) may be useful, but the datasets before the earthquake are not available. Third, we were unable to distinguish between the earthquake-related stress and other social stressors due to limitations in data availability. Fourth, the dose-response relationship between distance from the epicenter of the earthquake and incidence of PTB could support our findings. However, the sample size in Sichuan province is not large enough to allow us to conduct the dose-response analysis. Last, we only included secondary and tertiary hospitals in this study, which may introduce select bias and underestimate the associations. However, few pregnant women delivery in home or primary hospitals in China, and mostly due to exceptional circumstances [15, 37]. Considering all of the limitations mentioned above, the long-term risk of maternal exposure to Wenchuan earthquake on offspring birth outcomes should be interpreted with caution.

## Conclusions

Maternal exposure to the 2008 Wenchuan earthquake elevates the risk of PTB in offspring several years later after the earthquake, and only the male birth is vulnerable to the long-term effect. Maternal acute exposure to disasters could serve as a major monitor for long-term reproductive outcomes, and more research is needed to understand the underlining reasons for long-term disaster-related adverse birth outcomes.

## Supplementary information

**Additional file 1: Table S1.** Associations of maternal exposure to Wenchuan earthquake with adverse birth outcomes among all births of local pregnant women. **Table S2.** Associations of maternal exposure to Wenchuan earthquake with adverse birth outcomes among all livebirths of local pregnant women.

## Data Availability

The datasets used and/or analysed during the current study available from the corresponding author on reasonable request.

## Abbreviations

CEM: Coarsened exact matching
PTB: Preterm birth
LBW: Low birthweight
SGA: Small for gestational age
CLDS: China Labor and Delivery Survey
DCC: Data coordination center
WHO: World Health Organization
BMI: Body mass index
aRR: Adjusted risk ratio
CI: Confidence interval
