# Supplementary material for: Maternal exposure to Wenchuan earthquake and prolonged risk of offspring birth outcomes: a natural experiment study
Source: BMC Pregnancy Childbirth. 2020 Sep 22;20:552. doi: 10.1186/s12884-020-03206-1 (PMC7510090; doi:10.1186/s12884-020-03206-1)
Supplement: Supplementary file 1 — Additional file 1: Table S1. Associations of maternal exposure to Wenchuan earthquake with adverse birth outcomes among all births of local pregnant women. Table S2. Associations of maternal exposure to Wenchuan earthquake with adverse birth outcomes among all livebirths of local pregnant women. [file 12884_2020_3206_MOESM1_ESM.docx]

eTable1. Associations of maternal exposure to Wenchuan earthquake with adverse birth outcomes among all births of local pregnant women

| Birth outcome | Adjusted Risk Ratio^*^ (95% Confidence limits, P value) | |
| --- | --- | --- |
|  | Model 1 (All births) | Model 2 (Singleton births) |
| Total |  |  |
| Stillbirth | 1.18(0.69-2.01, 0.582) | 1.26(0.73-2.19, 0.409) |
| Preterm birth | 1.25(1.04-1.48, 0.014) | 1.22(1.01-1.47, 0.042) |
| Low birth weight | 1.07(0.88-1.30, 0.520) | 1.02(0.82-1.27, 0.850) |
| Small for gestational age | 0.91(0.69-1.20, 0.497) | 0.89(0.66-1.19, 0.438) |
| Girls |  |  |
| Stillbirth | 1.07(0.53-2.14, 0.857) | 1.20(0.58-2.49, 0.623) |
| Preterm | 1.10(0.84-1.43, 0.510) | 1.07(0.80-1.44, 0.631) |
| Low birth weight | 0.99(0.74-1.32, 0.947) | 0.96(0.70-1.32, 0.815) |
| Small for gestational age | 0.83(0.53-1.30, 0.420) | 0.82(0.51-1.32, 0.413) |
| Boys |  |  |
| Stillbirth | 1.26(0.57-2.80, 0.564) | 1.29(0.58-2.89, 0.538) |
| Preterm | 1.37(1.09-1.72, 0.006) | 1.34(1.05-1.71, 0.019) |
| Low birth weight | 1.15(0.87-1.51, 0.318) | 1.08(0.80-1.46, 0.602) |
| Small for gestational age | 1.02(0.83-1.25, 0.867) | 0.99(0.80-1.23, 0.942) |

^*^Adjusted maternal diseases, history of PTB, method of conception, parity, multiple pregnancy or singleton pregnancy, mode of delivery and sex of newborns.

eTable2. Associations of maternal exposure to Wenchuan earthquake with adverse birth outcomes among all livebirths of local pregnant women

| Birth outcome | Adjusted Risk Ratio^*^ (95% Confidence limits, P value) | |
| --- | --- | --- |
|  | Model 1 (All livebirths) | Model 2 (Singleton livebirths) |
| Total |  |  |
| Preterm birth | 1.27(1.05-1.52, 0.012) | 1.24(1.01-1.51, 0.037) |
| Low birth weight | 1.08(0.87-1.33, 0.483) | 1.01(0.80-1.28, 0.919) |
| Small for gestational age | 0.89(0.67-1.18, 0.427) | 0.87(0.64-1.17, 0.344) |
| Girls |  |  |
| Preterm | 1.08(0.81-1.43, 0.593) | 1.06(0.77-1.45, 0.731) |
| Low birth weight | 0.98(0.72-1.33, 0.891) | 0.93(0.66-1.29, 0.659) |
| Small for gestational age | 0.83(0.52-1.32, 0.436) | 0.81(0.50-1.32, 0.397) |
| Boys |  |  |
| Preterm | 1.44(1.14-1.81, 0.002) | 1.41(1.10-1.81, 0.007) |
| Low birth weight | 1.20(0.90-1.59, 0.212) | 1.12(0.82-1.53, 0.483) |
| Small for gestational age | 0.98(0.79-1.21, 0.823) | 0.95(0.76-1.18, 0.623) |

^*^Adjusted maternal diseases, history of PTB, method of conception, parity, multiple pregnancy or singleton pregnancy, mode of delivery and sex of newborns.
